# A systematic comparison of copy number alterations in four types of female cancer

**DOI:** 10.1186/s12885-016-2899-4

**Published:** 2016-11-22

**Authors:** Fatemeh Kaveh, Lars O. Baumbusch, Daniel Nebdal, Anne-Lise Børresen-Dale, Ole Christian Lingjærde, Hege Edvardsen, Vessela N. Kristensen, Hiroko K. Solvang

**Affiliations:** 1 Department of Genetics, Institute for Cancer Research, Oslo University Hospital Radiumhospitalet, Oslo, Norway; 2 Medical Genetics Department, Oslo University Hospital Ullevål, Oslo, Norway; 3 Department of Pediatric Research, Division of Pediatric and Adolescent Medicine, Oslo University Hospital Rikshospitalet, Oslo, Norway; 4 Department of Computer Science, University of Oslo, Oslo, Norway; 5 Department of Clinical Molecular Biology (EpiGen), Medical Division, Akershus University Hospital, Lørenskog, Norway; 6 Marine Mammals Research Group, Institute of Marine Research, Bergen, Norway

**Keywords:** Breast cancer, Cervical cancer, Copy number alteration, Endometrial cancer, Female cancers, Genomic Identification of Significant Targets in Cancer, Ovarian cancer

## Abstract

**Background:**

Detection and localization of genomic alterations and breakpoints are crucial in cancer research. The purpose of this study was to investigate, in a methodological and biological perspective, different female, hormone-dependent cancers to identify common and diverse DNA aberrations, genes, and pathways.

**Methods:**

In this work, we analyzed tissue samples from patients with breast (*n* = 112), ovarian (*n* = 74), endometrial (*n* = 84), or cervical (*n* = 76) cancer. To identify genomic aberrations, the Circular Binary Segmentation (CBS) and Piecewise Constant Fitting (PCF) algorithms were used and segmentation thresholds optimized. The Genomic Identification of Significant Targets in Cancer (GISTIC) algorithm was applied to the segmented data to identify significantly altered regions and the associated genes were analyzed by Ingenuity Pathway Analysis (IPA) to detect over-represented pathways and functions within the identified gene sets.

**Results and Discussion:**

Analyses of high-resolution copy number alterations in four different female cancer types are presented. For appropriately adjusted segmentation parameters the two segmentation algorithms CBS and PCF performed similarly. We identified one region at 8q24.3 with focal aberrations that was altered at significant frequency across all four cancer types. Considering both, broad regions and focal peaks, three additional regions with gains at significant frequency were revealed at 1p21.1, 8p22, and 13q21.33, respectively. Several of these events involve known cancer-related genes, like *PPP2R2A, PSCA, PTP4A3,* and *PTK2*. In the female reproductive system (ovarian, endometrial, and cervix [OEC]), we discovered three common events: copy number gains at 5p15.33 and 15q11.2, further a copy number loss at 8p21.2. Interestingly, as many as 75% of the aberrations (75% amplifications and 86% deletions) identified by GISTIC were specific for just one cancer type and represented distinct molecular pathways.

**Conclusions:**

Our results disclose that some prominent copy number changes are shared in the four examined female, hormone-dependent cancer whereas others are definitive to specific cancer types.

**Electronic supplementary material:**

The online version of this article (doi:10.1186/s12885-016-2899-4) contains supplementary material, which is available to authorized users.

## Background

In Norway, cancers of the breast and reproductive organs, including the cervix, ovaries, uterus (endometrium), fallopian tubes, vagina, and vulva, account for more than 34% of all cancers affecting women [1]. Breast, ovarian, cervical, and endometrial cancers are all associated with hormonal imbalance [2, 3]. Further, more than 99% of all cervix carcinomas are reported positive for infection with high risk human papillomavirus (HPV) [4]. Common characteristics of cancer cells are their abnormal proliferation, increased growth rate, and spreading to other organs [5]. Genomic alterations, including chromosomal rearrangements, copy number changes, and nucleotide substitutions, are regarded as fundamental cellular disruptions of almost all cancers [6, 7]. Although the genomic architecture varies considerably between cancer types, some genomic regions are commonly affected in several types, suggesting that some general mechanisms for selection are present. For example, aberrations of the tumor suppressor gene *PTEN*, located on 10q23, have been reported in various human malignant tumors, including endometrial, ovarian, breast, cervical, and lung cancer [8–10]. Detection of such aberrations may point to genes that are critical in cancer development and may point to targetable pathways [11]. Oligonucleotide arrays allow the detection of copy number alterations (CNAs) with high resolution on a genome-wide scale [12, 13]. Previous studies have identified many regions with known oncogenes, including *ERBB2* and *EGFR*, as well as tumor suppressor genes such as *TP53* [14].

In this study, copy number data from a total of 346 tumors from patients with breast (B), ovarian (O), endometrial (E), or cervical (C) cancers were analyzed with the aim of detecting similarities and differences between copy number changes of different female cancers. For segmentation of the copy number data, the test-based Circular Binary Segmentation (CBS) algorithm [15] and the penalized regression based Piecewise Constant Fitting (PCF) algorithm [16] were applied. After segmentation, the Genomic Identification of Significant Targets in Cancer (GISTIC) algorithm [17] was used to identify regions significantly altered in the different cancer data sets. These regions were further analyzed, on both the gene and pathway level, to reveal mechanisms of disease evolution common to multiple female cancer types. Taken together, these results may bring novel insight into the characteristics of the onset and progression of female cancers and possibly identify some common underlying mechanisms of hormonal influence in the risk of cancer.

## Methods

### Materials

We analyzed four different datasets of copy number alterations in tumors from patients with breast (*n* = 112 samples, *p* = 109315 probes), ovarian (*n* = 74 samples, *p* = 17984 probes), endometrial (*n* = 84 samples, *p* = 114782 probes), and cervical (*n* = 76 samples, *p* = 260531 probes) cancers. A summary of the clinicopathological characteristics for the investigated breast and ovarian cohorts is shown in Additional file 1: Table S1.

#### Breast

The 112 breast cancer samples are a subset of a larger patient series consisting of 920 samples collected from breast cancer patients to study the effect of disseminating tumor cells to the blood and bone marrow [18]. The samples were collected at five different hospitals in the Oslo region. This cohort has been extensively studied at both clinical and molecular level [18, 19]. Tumors were genotyped using the Human-1 109K BeadChip array (Illumina, San Diego, CA, USA). For each sample, the corresponding log R ratio (LRR) was extracted from two-channel allelic intensity values using Illumina's BeadStudio genotyping software [20].

#### Ovarian

The ovarian cohort, diagnosed and treated at the Department of Gynecological Oncology at the Oslo University Hospital the Norwegian Radium Hospital during the period May 1992 to February 2003, consisted of 74 patients diagnosed with serous ovarian cancers on routine pathology reports [21]. All patients had primary surgery, followed by adjuvant platinum-based chemotherapy. Copy number profiles of all samples were obtained with the Stanford 42k cDNA aCGH platform.

#### Endometrial

A total of 84 endometrial carcinomas data of 100K SNP Affymetrix Human Mapping 50K Xba and Hind arrays were selected from Gene Expression Omnibus (GEO, Series GSE14860). The samples were collected from 2001 to 2003 and primary tumor tissues were snap-frozen during hysterectomies. Genotyping was performed by Affymetrix Genotyping Tools Version 2.0. DNA-Chip Analyzer (dChip) software (www.dchip.org) to normalize probe-level signal intensities and data preprocessing [22]. Data were merged from the platforms, interlacing the markers according to position on the genome. Data were normalized and log2-transformed.

#### Cervix

The cervical carcinomas copy number data of 76 patients using 250K_Nsp SNP arrays were obtained from GEO (Series GSE10092) after exclusion of seven normal samples, eight duplicated samples, and nine cell lines. Data sets were evaluated at CUMC, Instituto Nacional de Cancerologia (Santa Fe de Bogota, Colombia) (Pulido et al., 2000), and the Department of Gynecology of Campus Benjamin Franklin, Charité Universitätsmedizin Berlin (Germany) [23]. We loaded the CEL-files to PennCNV software tool to obtain the Copy Number Variations (CNVs) from SNP genotyping array [24]. CEL files were sourced through the Mapping 250k Nsp genome information hg18. The raw signal intensities were normalized and log2-transformed.

### Methods

#### Copy number segmentation

Various segmentation methods exist for copy number data [25]. Here, the widely used CBS algorithm [15] and the more recent PCF algorithm [16] were applied. Briefly, CBS is a modified version of binary segmentation that splits chromosomes into contiguous regions based on a maximum t-statistic estimated by permutation. PCF fits a piecewise constant function to the data and for a given number of segments the method determines the optimal segmentation in a least squares sense. Both methods allow the trade-off between sensitivity and specificity to be controlled by the user (using the significance level for accepting a change point (α) in CBS and the penalty parameter (γ) in PCF). A range of adjustments for the trade-off were considered in order to explore short-range and long-range features in the copy number data, as well as to calibrate the performance of the two segmentation methods relative to each other. (For details about the algorithms and the calibration, see Additional file 2: Supplementary Methods).

#### GISTIC

To distinguish biologically significant copy number changes from random events, we applied GISTIC 2.0 [26]. GISTIC requires segmented data. In this article, we segmented data applying the CBS or PCF algorithms. Location annotations were based on hg18. First, GISTIC calculates a G-score associated with the amplitude of the aberration and the frequency of incidence in multiple tumors. Second, the G-scores is assessed significance by q-value based on permutations of the locations of the copy number segments in the tumors; thus, the level of significant q-values is calculated for each observed region. Only alterations that surpass a specified q-value threshold are identified as being significant [11, 17]. Regions with a log2 ratio above a threshold value (Default = 0.1) are considered being amplified and regions with a log2 ratio below a negative threshold (Default = 0.1) are considered being deletions. Focal events are regions of repeated genetic information that span over not more than 25% of the chromosome arm. All regions greater than that limit are termed broad. The broad regions (arm-level significance) are computed by comparing the frequency of gains or losses of each arm to the expected rate given its size [11, 15, 26].

#### Capturing consistent GISTIC output

Depending on the selection of the segmentation method and the trade-off for sensitivity-specificity balance, the number of segments and their precise boundaries may vary. Some deviation in the result of GISTIC is expected; however, a consistent output of GISTIC is required for the correct biological interpretation of the data. To test the consistency of the GISTIC output for data segmented by different algorithms, in this case CBS or PCF, the combination of CBS + GISTIC or PCF + GISTIC was applied to simulated and real data and a range of different values for the sensitivity-specificity parameter *α* in CBS and γ in PCF were explored (see Additional file 2: Supplementary Methods and Additional file 3: Tables S2 and Additional file 4: S3). To achieve an optimal threshold for α and γ in each data set, we identified a consistent number of GISTIC focal peaks and confirmed whether these peaks generated by CBS-segmentation highly overlapped with peaks based on PCF-segmentation (Additional file 5: Table S4).

#### Visualization of copy number changes

Following identification of the significant copy number changes, we used the software package of Circos (http://circos.ca) to visualize the genomic localization and rearrangements [27].

#### Pathway and network analysis

Ingenuity Pathway Analysis (IPA) (http://www.ingenuity.com; version 9.0; release date: 2012-08-11, content version: 14197757, build: 172788) was used to analyze selected sets of genes in order to identify over-represented canonical pathways and biological functional interactions. The IPA core analysis module allows detection of interactions between genes and proteins, related networks, functions, and canonical pathways in the context of biological processes. Gene sets identified by GISTIC were uploaded into IPA for further analysis. The only filter criteria used for the network analysis was “only consider molecules and/or relationships where species = Human”. Both, direct and indirect relationships, as well as endogenous chemicals were taken into account and for the network analysis the maximum number of molecules allowed per network was set to 140. The significance of the association between the cancer gene sets was assessed by the False Discovery Rate (FDR) [28].

## Results

### Comparing different segmentation algorithms

**Fig. 1:**
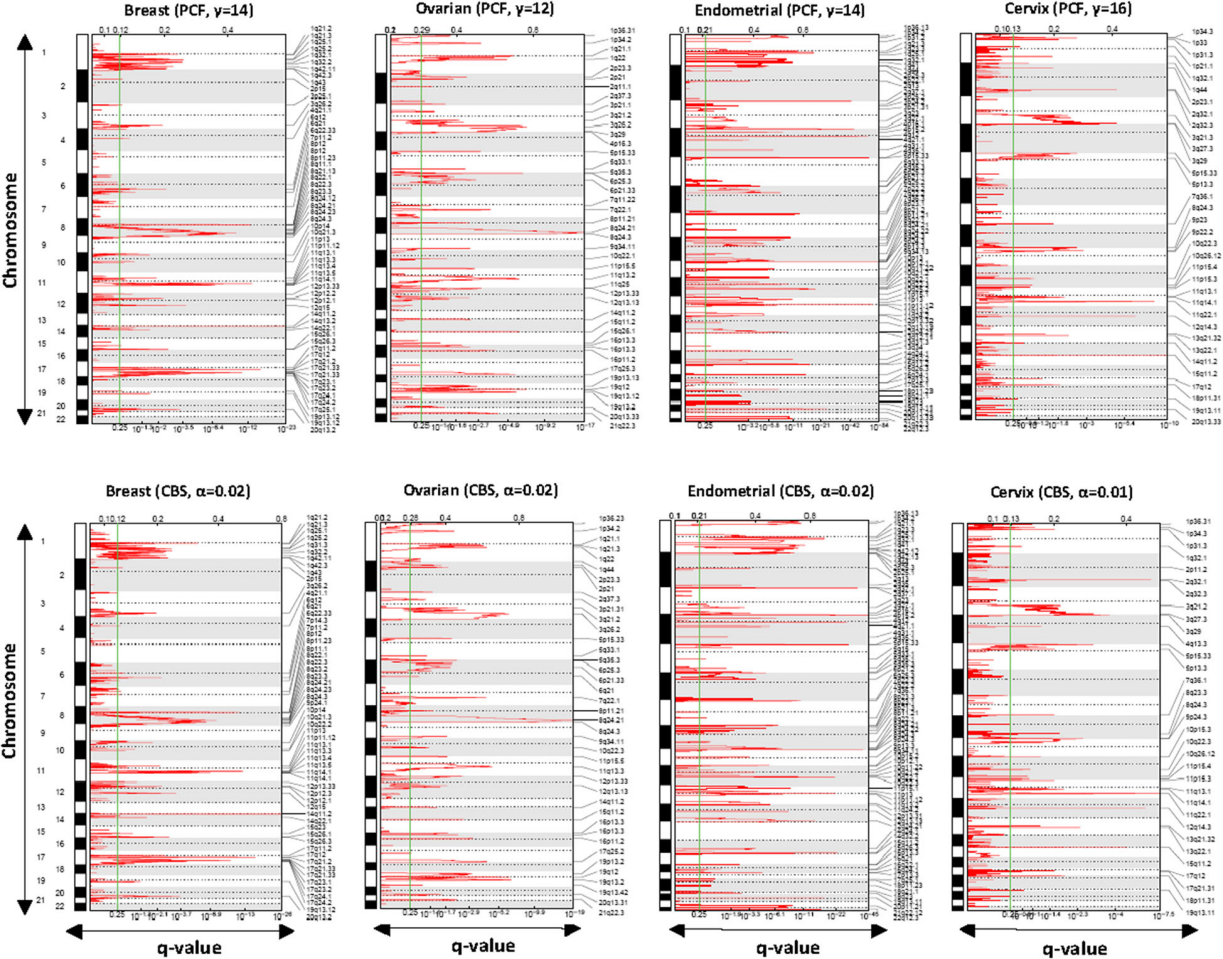
Circular Binary Segmentation (CBS) - and Piecewise Constant Fit (PCF) - segmented data (amplifications). Significant copy number alterations (gains, colored in *red*) are illustrated in four different cohorts; breast, ovarian, endometrial, and cervical cancers, determined by two different segmentations algorithms PCF and CBS. Both methods allow the trade-off between sensitivity and specificity to be controlled by the user using the significance level for accepting a change point (α) in CBS and the penalty parameter (γ) in PCF. We selected γ = {14, 12, 14, and 16} for the PCF-segmentation and α = {0.02, 0.02, 0.02, and 0.01} for the CBS-segmentation. The statistical significance of the aberrations is displayed as FDR q-values to account for multiple-hypothesis testing (x-axis). Chromosome positions are indicated alongside the y-axis with centromere positions indicated by dotted lines. The significance threshold is allocated by a *green* line

**Fig. 2:**
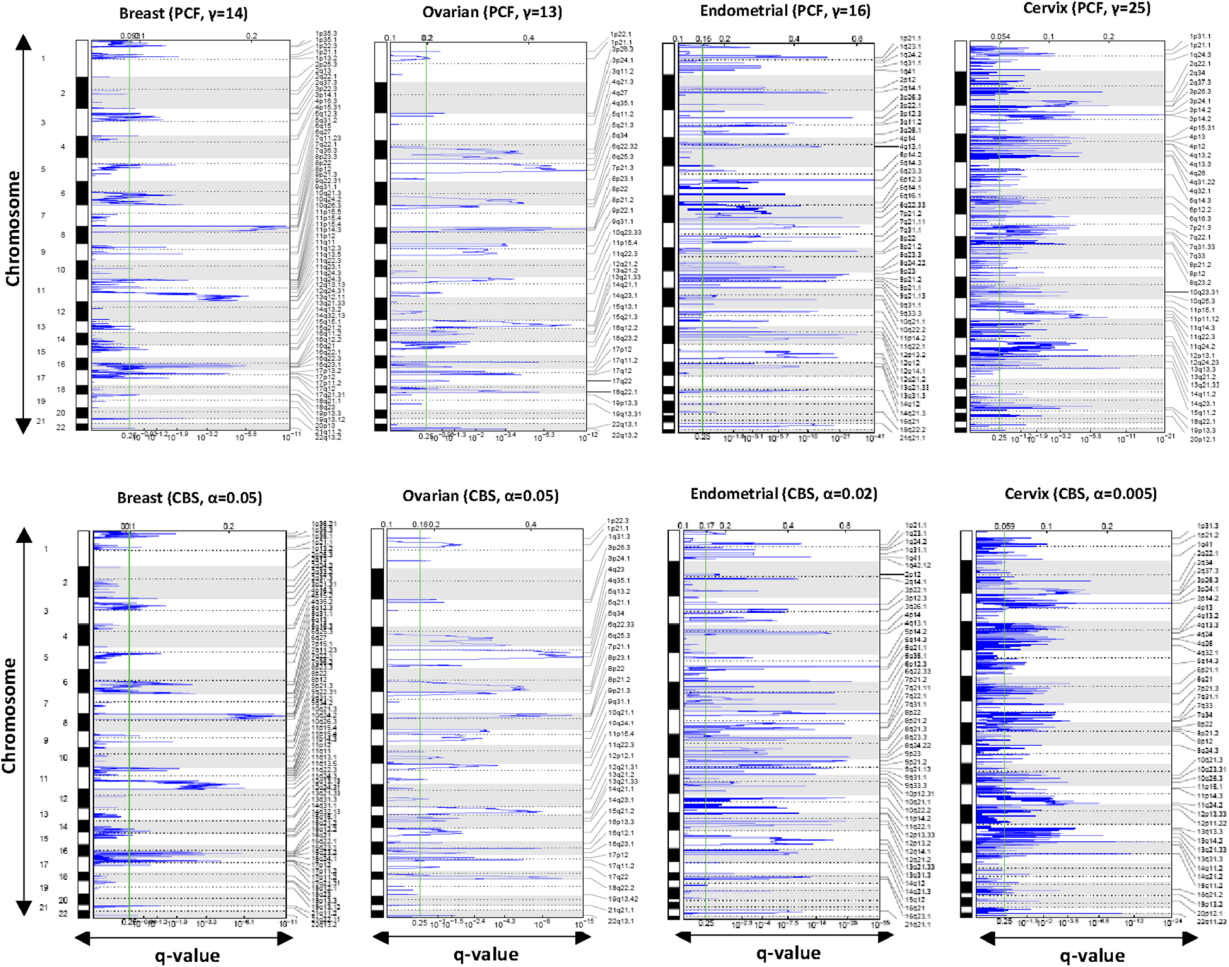
CBS- and PCF-segmented data (deletions). Significant copy number alterations (deletion, colored in *blue*) are shown in four different cohorts; breast, ovarian, endometrial, and cervical cancers, analyzed by the two different segmentations algorithms PCF and CBS. For both methods, the threshold for calling copy number gains and losses can be adjusted. For PCF-segmentation, γ = {14, 13, 16, and 25} was selected and for CBS-segmentation α = {0.05, 0.05, 0.02, and 0.005} was defined. The X-axis indicates the statistical significance of the aberrations (q-values). Chromosome positions are indicated alongside the y-axis with centromere positions designated by dotted lines. The significance threshold is allocated by a *green* line

Accurate detection of chromosomal aberrations is crucial for comparing multiple CNA data sets originating from different platforms and cancer types. The performance of CBS- and PCF-segmented data as input for GISTIC were compared using both simulated and real data from tumor samples from patients with breast (B), ovarian (O), endometrial (E), and cervical (C) cancers, hereafter denoted as BOEC (Additional file 3: Table S2 and Additional file 4: Table S3). For both methods, the threshold for calling copy number gains and losses can be adjusted and must be set appropriately. In most publications, the default values of α and γ are used [29, 30], but as shown here, variations in these parameters may influence the results substantially and the optimal γ and α should be adjusted for every dataset. During the segmentation process, the CBS algorithm illustrated slower processing than PCF. To determine α and γ, we compared the significant regions identified by GISTIC (for details see Additional file 2: Supplementary Methods) for various choices of α and γ and selected the parameter values that maximized the overlap between the GISTIC outputs for the two methods. Detection of amplification events was consistently less dependent on the segmentation procedure than that of deletion events in the different cancers. A large fraction of amplifications (80-86%) and deletions (58–84%) were detected by GISTIC after segmentation by both methods (Additional file 5: Table S4). The significant aberrations fall into two types, focal and broad (as described in Material and Methods). We observed that PCF-segmented data produced a higher number of GISTIC focal peaks (Additional file 6: Figure S1). Based on the adjustment among different arrays, optimal α and γ were selected separately for each data set (Figs. 1 and 2). In each cohort, the numbers of focal events surpassing the significance threshold (green line in Figs. 1 and 2) together with the locations of the peak regions have been identified (Additional file 7: Table S5).

### Loci of specific amplifications and deletions according to GISTIC

**Fig. 3:**
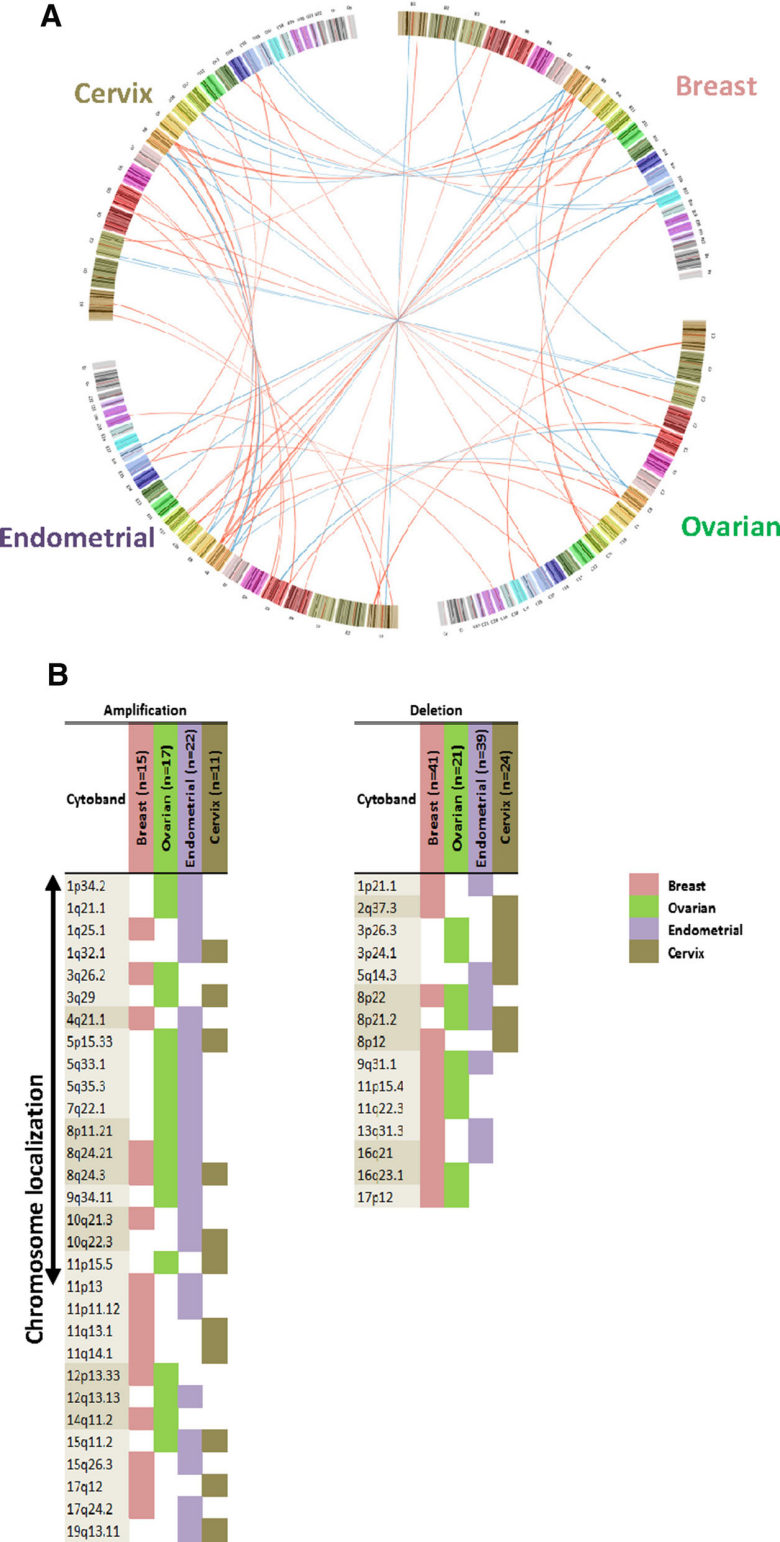
GISTIC focal peaks for overlapping regions of CBS- and PCF-segmented data in four different female cancers. Circos-plot of GISTIC copy number alteration data obtained from female patients with breast (B), ovarian (O), endometrial (E), or cervical (C) cancers. The breast (*pink*), ovarian (*green*), endometrial (*purple*), and cervical (*brown*) cancers are presented in a clockwise direction. For each cohort, from the top of circle, chromosomes 1 – 23 are displayed and each chromosome’s cytoband is colored differently. Aberrations are represented by lines linking the overlapping cytobands between different cancers. The width of the lines is matched to the cytobands. Amplification lines are colored in *red* and deletion lines are marked in *blue*. Panel B demonstrates similarities and divergences between the four investigated female cancers for at least two genomic regions identified by the CBS and PCF algorithms. On the left side, copy number gains of GISTIC focal peaks are presented and on the right side are copy number loss of GISTIC focal peaks illustrated, for both, CBS- and PCF-segmented data. The number of peaks obtained by GISTIC, for breast, ovarian, endometrial, and cervical cancer that are colored in *pink*, *green*, *purple*, and *brown*, respectively

GISTIC was applied to the breast, ovarian, endometrial, and cervical sample sets to detect copy number changes associated with either single or multiple cancer types. The GISTIC focal peaks were compared to identify the shared altered genomic regions independently for amplification and deletion. To attain a robust estimate of the aberrant regions, the GISTIC output was analyzed separately for each segmentation algorithm (Additional file 7: Table S5 and Additional file 8: Figure S2). Using CBS-segmented input data for GISTIC, we identified a total of 404 significant regions of focal aberrations including 124 regions in breast (*n* = 57 amplifications and *n* = 67 deletions), 79 regions in ovarian (*n* = 42 amplifications and *n* = 37 deletions), 124 regions in endometrial (*n* = 74 amplifications and *n* = 50 deletions), and 77 regions in cervical cancers (*n* = 32 amplifications and *n* = 45 deletions) (Additional file 9: Figure S3). Applying PCF-segmented input data for GISTIC, we observed a total 402 significant regions that consisting of 122 regions in breast (*n* = 58 amplifications and *n* = 64 deletions), 80 regions in ovarian (*n* = 42 amplifications and *n* = 38 deletions), 123 regions in endometrial (*n* = 74 amplifications and *n* = 49 deletions), and 77 regions in cervical cancers (*n* = 33 amplifications and *n* = 44 deletions) (Additional file 7: Table S5 and Additional file 10: Figure S4). These results indicate that GISTIC output is most stable after optimizing the segmentation parameters *α* in CBS and γ in PCF. Overlapping focal peaks between all cancers pair-wise are summarized in Fig. 3a and Additional file 11: Table S6. Using theses stringency levels, the majority of the identified regions were specific for only one cancer type with 75% (89/112) of the amplified and 86% (92/107) of the deleted regions (Fig. 3b). However, one CNA event was common for all studied female cancer types, a copy number gain at 8q24.3.

Including broad peak regions into the analysis, additional common events for all studied cancers were detected at 1p, 1q, 13q, 17p, 20p, 20q, and 22q (Additional file 12: Table S7). Further, for cancers in the female reproductive system (ovarian, endometrial, and cervix [OEC]), we identified three common incidences; two copy number gains at 5p15.33 and 15q11.2, and one copy number loss at 8p21.2 (Fig. 3b).

### Genes residing in loci of specific amplifications and deletions

**Fig. 4:**
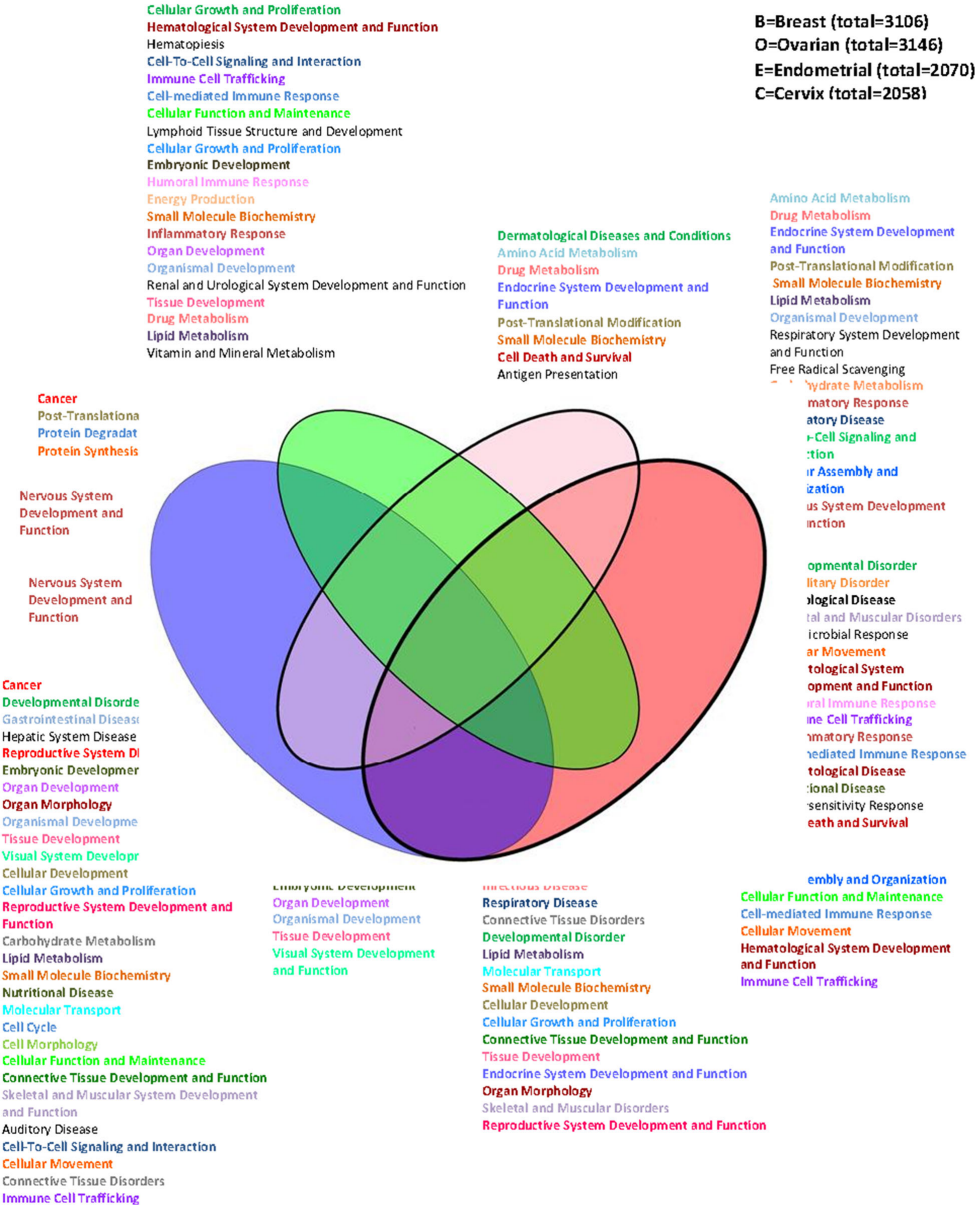
Overlap between gene sets of four female cancer– Top biological functions. The Venn diagram displays joint genes identified by both, CBS and PCF algorithms located within the regions identified by GISTIC. The total number of genes for each data set is presented on the top right panel. Top biological functions and top canonical pathways for each region of the overlapped cancers are stated

Genes classified by both algorithms and located within the broad or focal peak regions identified by GISTIC (Additional file 13: Table S8) were extracted and the deregulated genes for each cancer type are reported. We obtained 3106 genes for breast, 3146 genes for ovarian, 2070 genes for endometrial, and 2058 genes for cervical cancer. The degree of overlap between these lists is visualized in a Venn diagram (Fig. 4). The number of identified common genes was 235 for endometrial and ovarian (EO), 259 for breast and endometrial (BE), 285 for breast and cervix (BC), 87 for ovarian and cervix (OC), 164 for endometrial and cervix (EC), and 461 for breast and ovarian (BO) cancers. Further, shared genes among three cancer types, we found 128 for endometrial, ovarian, and cervix (EOC), 106 for breast, ovarian, and cervix (BOC), 50 for breast, endometrial, and cervix (BEC), and 20 for breast, ovarian, and endometrial (BOE) cancers. Two genes, actin-organizing protein *KLHL1* at 13q21.33 and *COL11A1 (collagen, type XI, alpha 1)* at 1p21.1 were detected as joint deletions in all four cancer types (breast, ovarian, endometrial, and cervical, BOEC) (Fig. 4 and Additional file 14: Figure S5).

### Pathways deregulated by significant DNA aberrations in female cancers

**Fig. 5:**
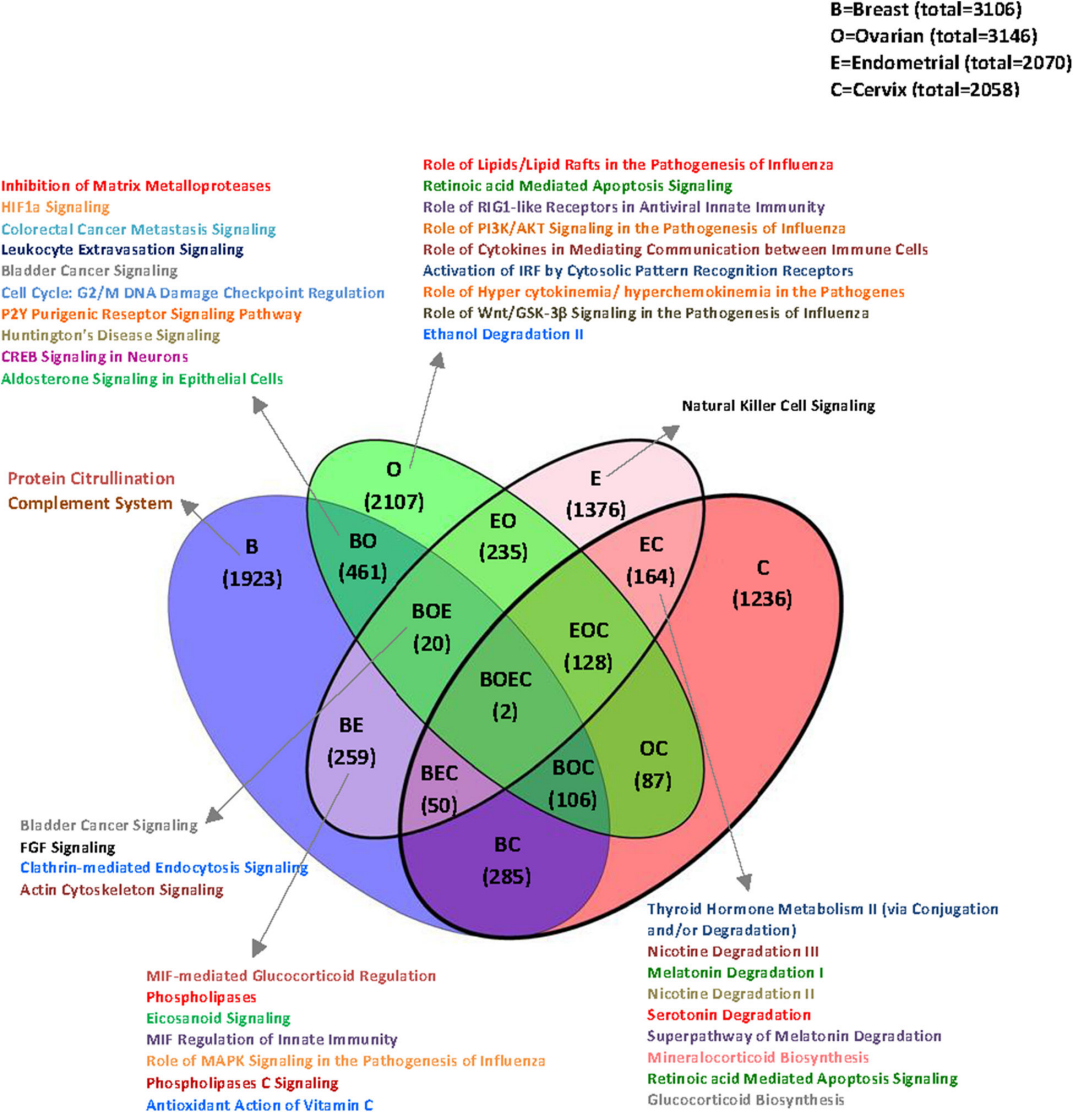
Overlap between gene sets of four female cancers – Top canonical pathways. The Venn diagram illustrates the joint genes identified by both CBS and PCF algorithms, located within the regions identified by GISTIC. The total number of genes for each data set is exhibited on the top right panel. Top canonical pathways, at 5% FDR, for each region of the joint cancers are displayed

Genes located in regions identified by GISTIC analysis (Additional file 13: Table S8) were submitted to the IPA software to investigate whether these genes are organized in combinatorial pathways. IPA was first attained separately for each cancer data set (Additional file 15: Table S9). For the breast cancer gene lists (*n* = 3106), IPA resulted in solely one top biological function, “nervous system development and function”. Genes aberrant in ovarian cancer (*n* = 3146) shared pathways like “cellular development, cell-to-cell signaling and interaction, cellular function and maintenance, cellular growth and proliferation, and immune cell trafficking and other inflammatory response signatures” (Fig. 4). At 5% FDR, the IPA analysis exhibited the most significant canonical pathways (Fig. 5 and Additional file 16: Table S10), including “protein citrullination” and “complement activation for the breast cancer aberrated genes”, whereas eight significant canonical pathways were discovered for ovarian cancer such as: “role of lipids/lipid, retinoic acid mediated apoptosis signaling, role of *RIG1*-like receptors in antiviral innate immunity, activation of *IRF* by cytosolic pattern recognition receptors, and role of *PI3K*/*AKT* signaling in the pathogenesis of influenza”. The single significant canonical pathway at 5% FDR for cervical cancer associated genes was “thyroid hormone metabolism II (via conjugation and/or degradation)” (Additional file 15: Table S9) and for endometrial cancer “Natural Killer cell signaling”. Although unique lists of genes for each of the sections of the Venn diagram were studied, in the end there turned-up similar overlapping genes between different cancer types (Fig. 4), which were also uploaded to IPA for the assignment of biological functions as well as for identifying the most significantly associated canonical (curated) pathways. The most frequent biological functions among all genes and all studied cancers were lipid metabolism, small molecule biochemistry, cellular growth and proliferation, cellular development, and post-translational modification.

## Discussion

In the last decade, the genomic profiles of tumors of many different tissues have been analyzed. Especially for tumors originating from female breast or the reproductive organs common copy number gains or losses have been observed [31–35]. However, despite this obvious coincidence of genetic traits, to our knowledge, so far no systematic comparison has been performed to identify universal or cancer-type specific regions and genes in female cancers. The reasons for that may be manifold, including the limited number of samples for specific cancer types, systematic tissue-dependent differences (like ovarian tumors, that are often detected at a later stage with on average larger sizes), and the lack of available analytic methods taking care of the challenges generated by combining data originating from different array platforms. Baumbusch et al. (2008) compared different platforms and illustrated that despite the consistency of platforms, specific variations in frequency are visible in the studied platforms [29]. We do not directly quantify the amplitudes but compared the frequencies of different platforms for each tumour type. Here in this study, we chose a rather strict analysis pipeline to avoid too many false positive results without losing infrequent regions and genes. It is important to detect regions and genes common for breast, ovarian, endometrial and cervical cancers; however, it may be even more interesting to identify regions and genes unique for the various cancer types in order to reveal underlying mechanisms of disease genesis and progression in female cancers.

Accurate detection of chromosomal aberrations is crucial for comparing multiple CNA datasets originating from different platforms and cancer types is dependent on an accurate segmentation algorithm matching an optimal level to adjust for platform- and tissue-specific variations. Different algorithms for aCGH analysis have been compared and described previously [25]. Depending on the segmentation algorithm (and the chosen significant levels) the identified copy number gains and losses may vary in their occurrence, number, and distribution. From the variety of available methods for analyzing CNA we chose CBS, as one of the most commonly used algorithms, and PCF, a novel platform independent, efficient, and flexible algorithm. The two segmentation methods were tested separately for each platform to assess multiple segmented data generated by several variables of the parameters and to identify a consistent and adjusted threshold among different arrays and to detect an optimal segmentation parameter for our comparative analysis. We found high similarities between the two segmentation algorithms. In this paper, we simultaneously searched the optimum of both parameters related to two segmentation algorithms; however, we could consider the consistency to the GISTIC output by other segmentation methods or another algorithm, like fixing one parameter of an algorithm and searching the optimum parameter of the other algorithms.

For both methods the threshold for calling copy number gains and losses in different arrays can be adjusted and must be set properly. In most publications, the default values of α and γ [29, 30] are used but, as shown here, varying these parameters may substantially influence the results and we recommend to adjust and optimize γ and α for each dataset. The selection of an appropriate value is hence important and mostly depends on the number of probes in each data set and the level of noise.

Our study represents analysis of high-resolution copy-number profiles of various female cancers. This analysis shows a strong tendency for significant focal aberrations in some regions of female cancers. Significant events of genomic amplification were more often detected by both, segmentation procedures in all types of arrays, (over 82%) consistency between the arrays, which was much more than what was found for deletions; may be explained by the possibility of only two copies allowed for deletions.

Previous studies of copy-number alterations have focused on one or two cancer types, such as breast and ovarian. Mutations in *BRCA1* and *BRCA2* genes confer a high risk of both breast and ovarian cancer [36]. Cheng et al. (2006) have reported gene *Rab25*, located on 1q22, as a potential driver of ovarian and breast tumor development [37]. We identified this candidate gene (*Rab25*) in the altered regions (gain on chromosome 1q) of endometrial and ovarian cancers. Another genetic event seen in both breast and ovarian cancer is loss of heterozygosity (LOH) on the short arm of chromosome 8 [38, 39]. We have previously shown that genes in these regions such as 8q24.13 and 8p23.2 are affected by a non-random loss of heterozygosity in breast cancer [40].

Recent whole genome association studies of common epithelial cancers have revealed that the most prevalent gains are detected at the 8q arm [41]. It was also the single event common to all female cancers studied in this paper. This locus is also the one with most commonly identified susceptibility SNPs by GWAS for different cancers. Among the 97 annotated genes found affected by chromosomal focal gain event in 8q24.3 in this study, we recognized some susceptibility genes that have previously been reported associated to risks of different cancer types. For instance, the genetic variation in prostate stem cell antigen (*PSCA*) gene has been associated with the risk of bladder cancer, pancreatic cancer, and prostate cancer in multiple GWAS studies [42, 43]. Additionally, recent GWAS studies have shown that two single-nucleotide polymorphisms (SNPs) in *PSCA* gene are associated with gastric cancer [44]. Hao et al. (2011) have reported the *PSCA* expression in invasive micropapillary carcinoma of breast [45]. *CYP11B2* residing in the same region has been associated with adrenocortical tumor development [46]. The *PTK2* gene located on 8q24.3 is a member of the focal adhesion kinase (FAK) and has been suggested to be involved in early breast carcinogenesis [47]. This region also contains some cancer related genes such as *PTP4A3* and *PTK2* genes. The over expression of *PTP4A3* has been reported in liver metastases derived from colorectal cancer as well as breast cancer, ovarian cancer, gastric cancer, esophageal carcinoma, and invasive cervical cancer [48]. This gene promotes the cell invasion and activity by stimulating of Rho signaling pathways [49]. *PTK2* gene has been identified as a critical gene in breast carcinoma, too [50]. Ishikawa et al. (2007) have suggested the *LY6K* gene as a potential histochemical biomarker for lung and esophageal cancers and its potential activation role in cervical cancers [51]. Ambatipudi et al. (2012) also have shown the over expression of *LY6K* in gingivobuccal complex cancers [52]. Frequent increases in DNA copy number at the chromosomal region of 8q24.3 have been reported to serve as a prognostic marker in early stage ER+ breast cancers [53] and ovarian carcinomas [54]. This region is also frequently amplified in endometrial cancer [55] and contributes to the cancer risk in bladder cancer [56], colorectal cancer [57], adrenocortical tumor development [46], and gingivobuccal cancers, -a sub-site of oral cancer [52].

Among the female reproductive system (OEC) data sets, we distinguished two common gain events (5p15.33 and 15q11.2) and one loss event on chromosome 8p21.2. The copy number gain at 5p15.33 and the loss at 8p21.2 have been reported as potential predictive markers of drug-resistant phenotype in advanced serous ovarian cancer [54]. A number of studies have shown that the frequent gain at 5p15.33 in cervical cancer may play an important role beside the HPV infection [58, 59]. GWAS studies have shown an association between a variant at 5p15.33 region with the risk of many tumors including breast [60], testicular [61], bladder [62], lung [63], pancreatic [64], and glioma cancers [65] in genes such as *TERT* and *CLPTM1L* The *TERT* enzyme is a protein component of telomerase, a ribonucleoprotein polymerase that regenerates telomere ends by the addition of nucleotide repeat sequences [60]. A gene variant in the *TERT* gene has been suggested to be associated with epithelial ovarian cancer [60]. Another gene in 5p15.33 was *CLPTM1L* that is expressed in various cancer types, including lung and ovarian cancers. It plays an important role in the induction of apoptosis in cisplatin-sensitive cells [60]. Kersemekers et al. (1998) reported the presence of a tumor-suppressor gene on 15q11.2 [66]. The other region found altered in cancers of the reproductive system, 8p21.2 harbours the *BNIP3L* gene and has been identified as a tumor suppressor gene in breast cancer, ovarian cancer, and prostate cancer [39]. This gene encodes a protein that is homologous to the proapoptotic protein *BNIP3* and has the ability to suppress colony formation in soft agar. Curtis et al. (2012) have shown heterozygous and homozygous deletions of *PPP2R2A* gene are located on 8p21.2 in breast cancer [67].

In each of the four female cancers individually, we observed in the aberrant regions known driver genes. For example, in breast cancer, we identified divide according to amplified or deleted regions genes *TP53BP2, TP53INP1, K-RAS, BIRC5, TP53TG3, TP53I11, CCND1, FGFR1OP, ATM, PMS1, H-RAS, N-RAS*, and *MYCBP2. TP53BP2* gene encodes a member of the *ASPP* (apoptosis-stimulating protein of p53) family of p53 interacting proteins. Over-expression of *TP53INP1* has been reported in breast cancer as a potential prognostic marker [68]. Many of these proteins represent regulatory molecules including members of the p53 family that regulate apoptosis and cell growth through interactions with other regulatory molecules [69]. For example *BIRC5*, a member of the inhibitor of apoptosis (IAP) gene family, encodes negative regulatory proteins that prevent apoptotic cell death. Over expression of *BIRC5* gene has been reported to be correlated with loss of specific chromosomal regions in breast tumor cells [70]. Activating *K-RAS* gene point mutations have been detected in breast cancer [71]. *H-, K*-, and *N-RAS* genes are a subfamily of the huge *RAS/RHO/RAB* superfamily and encode ubiquitous cytoplasmic *GTP* binding p21 proteins involved in signal transduction [72]. In ovarian cancer, we detected some known cell cycle regulating driver genes such as *CSF1R*, *AKT2*, *FGF3*, *EEF1A2*, *MUC17*, *NOTCH2*, *CDKN2A*, *MYC*, and *ERBB2IP*. In endometrial cancer, we observed in the aberrant regions genes such as *ADIPOR1* which adiponectin levels have been shown to correlate with endometrial cancer risk [73]. *FOXC1* gene that screens of primary endometrial cancer have revealed that this gene is deleted in 6.7% out of 11.7% transcriptional silenced primary cancer and suggests that it functions as a tumour suppressor, *PAEP*, *THBS2*, and *PTK2*. In cervical cancer, genes *PTK2*, *DDK3*, *DLG1*, *MUC6*, and *HSPD1* have been reported to be over-expressed in exo-cervix cancer [72].

The actin-organizing protein *KLHL1* gene is one of the two identified common genes among all of the tested female cancers. The kelch-related proteins (KLHL) play an important role for the maintenance of the ordered cytoskeleton [74]. They have diverse functions in cell morphology, cell organization, and gene-expression; and form multi-protein complexes through contact sites in their β-propeller domains [75]. Alterations and mutations of these proteins have been reported in brain tumors and neurodegenerative disorders [74, 76]. *COL11A1, (collagen, type XI, alpha 1),* is essential for normal formation of cartilage collagen fibrils and the cohesive properties of cartilage and has been identified as a potential metastasis-associated gene in lung [77], oral cavity [78], and in cancer associated fibroblasts [79].

Interestingly, despite the little overlap of loci identified as significantly aberrant in the different cancer types, some common pathways were affected. Lipid metabolism appears on O, BOE, BOC, EC, and E cancers. Activation of lipid metabolism has been reported to be an early event in carcinogenesis [80] and many studies at the single-gene level of lipid metabolism have revealed an effect on tumor genesis [81]. Tissue development appears as affected in O, BOE, BEC, and BOC. Immune cell trafficking in O, BOE, EOC, and OC, inflammatory response in O, EOC, and EC are also affected. The most common deregulated pathway is bladder cancer signaling which observed between BO and BOE. On the contrary, pathways such as FGF Signaling, Clathrin-mediated Endocytosis Signaling, *HIF1* *A* Signaling, and etc. were only uniquely observed as deregulated in a specific cancer type.

## Conclusions

Recent technological advances in genome-wide analysis have made it possible to detect chromosomal aberrations leading to discovery of novel oncogenes among different cancers. However, previous studies have primarily focused on cancer of the same tissues. Here in this study, we compared cancer generated from different tissue types to identify the common and disperse regions, genes and pathways in female cancers. Technical challenges, like consistency across platforms, have been handled by adjusting simultaneously the segmentation thresholds in two different segmentation algorithms. However, we found five events common to all studied cancers, there is possibility to combine other segmentation algorithms to have robust regions.

## Additional files

Additional file 1: Table S1. Additional clinical data for the Breast and Ovarian cohort. A summary of the clinicopathological characteristics for the breast and ovarian cohorts is available (XLSX 102 kb)
Additional file 2: Supplementary methods (DOCX 398 kb)
Additional file 3: Table S2. GISTIC focal peaks for simulated data – gains and losses. Table S2A exemplifies the number of focal peaks of simulation data for PCF-segmented input data to GISTIC and variable γ from 10 to 90. Table S2B represents GISTIC focal peaks of simulated data for CBS-segmented input data to GISTIC and α varied from 0.00001 to 0.1. (XLSX 10 kb)
Additional file 4: Table S3. GISTIC focal peaks for female cancers – gains and losses. Table S3A shows the number of focal peaks for PCF-segmented input data to GISTIC and variable γ in a range of 10 to 90 for breast, ovarian, endometrial, and cervical cancers. Table S3B represents GISTIC focal peaks for CBS-segmented input data to GISTIC and variable α from 0.00001 to 0.1 for the above cohorts. (XLSX 12 kb)
Additional file 5: Table S4. Highest percentage of overlap between α (CBS) and γ (PCF) – gains and losses. Table S4A demonstrates the highest percentage of overlap between α (CBS) and γ (PCF) for amplification GISTIC focal peaks of female cancers. Table S4B shows the highest overlap of α and γ for deletion GISTIC focal peaks. (XLSX 12 kb)
Additional file 6: Figure S1. Genomic Identification of Significant Targets (GISTIC) outputs for Circular Binary Segmentation (CBS) - or Piecewise Constant Fit (PCF) - segmented input data. The number of peaks attained by GISTIC on the y-axis is plotted against the two changing parameters α for CBS and γ for PCF on the x-axis. GISTIC peaks of amplification applying CBS-segmented data are illustrated in pink and PCF-segmented data in red, respectively. Deletion peaks are colored in green for CBS-segmented input data and in blue for PCF-segmented data. From top to bottom are shown GISTIC focal peaks for breast, ovarian, endometrial, and cervical cancers, to the left for PCF-segmented input data (A, C, E, and G) and to the right for CBS-segmented input data (B, D, F and H), respectively. For further analysis are the selected α and γ highlighted with a colored square. (PDF 362 kb)
Additional file 7: Table S5. GISTIC focal peaks for selected α (CBS) and γ (PCF) - gains and losses. Table S5 reveals the GISTIC focal peaks of the selected values in Table S4. Indicated by asterisks (*) are the joint regions between cancer types for at least two cancers displaying a common genomic region, and (n) representing the number of events in each cohort. (XLSX 37 kb)
Additional file 8: Figure S2. Comparison of PCF and CBS results for detection of joint peaks in different female cancers. The number of peaks obtained by GISTIC are revealed for breast, ovarian, endometrial, and cervical cancers, colored in *pink*, *green*, *purple*, and *brown*, respectively. Panel A shows the amplification GISTIC focal peaks for PCF-segmented data and panel B for CBS-segmented input data. Panels C and D illustrate the GISTIC focal peaks for deletions for PCF- and CBS-segmented input data, respectively. (PDF 114 kb)
Additional file 9: Figure S3. Focal peaks of GISTIC for female cancers based on CBS-segmented data (amplifications and deletions). Panel A represents the aberrations of various female cancers for CBS-segmented input data to GISTIC in a circos-plot. In clockwise direction, breast (B, *pink*), ovarian (O, *green*), endometrial (E, *purple*), or cervical (C, *brown*) cancers are displayed. From the top of circles with 23 chromosomes presented for each cohort with each chromosome’s cytobands is colored differently. Aberrations are represented by lines linking the overlapping cytobands between the various female cancers. The width of lines is matched to the size of each cytoband. Amplification lines are colored in red and deletion lines are illustrated in blue. Panel B focuses separately on each chromosome. (PDF 329 kb)
Additional file 10: Figure S4. Focal peaks of GISTIC for female cancers based on PCF-segmented input data (amplifications and deletions). Panel A represents the aberrations of female cancers in a circular format for PCF-segmented input data to GISTIC. In clockwise direction, breast, ovarian, endometrial, and cervical cancers are displayed in *pink* (breast), *green* (ovarian), *purple* (endometrial), or *brown* (cervical) cancers. From top of the circles are displayed 23 chromosomes for each cohort, each chromosome’s cytobands colored differently. Rearrangements are represented by lines connecting the overlapping cytobands between the different female cancers. The width of lines is matched with the size of each cytoband. Amplification lines are colored in red and deletion lines in blue, respectively. Panel B focuses separately on each chromosome. (PDF 330 kb)
Additional file 11: Table S6. GISTIC focal peaks for joint regions of CBS and PCF gains and losses. This table shows the GISTIC focal peaks of the overlapping regions between CBS and PCF. (XLSX 15 kb)
Additional file 12: Table S7. Arm-level significant table for four studied cancers. Table S7 illustrates the significant arm-levels for the four different female cancer types. The colored arms show the significant ranges. Breast, ovarian, endometrial, and cervical cancers are colored in pink, green, purple, and brown, respectively. (XLSX 12 kb)
Additional file 13: Table S8. Genes residing in loci of specific amplifications and deletions (CBS and PCF) - gains and losses. **Table S8.** reveals the list of genes for the selected values of α (CBS) and γ (PCF). (PDF 41 kb)
Additional file 14: Figure S5. A schematic view of two common genes among female cancers. This schematic view shows the genomic positions of two genes that were found common among female cancers. The figure further suggests an explanation about the lack of any common genes for the focal aberrations at 8q24.3. (XLSX 493 kb)
Additional file 15: Table S9. Top networks, biological functions, and top canonical pathways (CBS and PCF) - gains and losses. Table S9 illustrates the IPA results including: The top five network functions, biological functions, and top canonical pathways. These results were obtained based on the gene lists (both gain and loss) of GISTIC focal peaks for input data based on both, CBS- and PCF-segmented data for each cancer type. Gray colored regions show the most significant functions/pathways at 5% FDR. (XLSX 15 kb)
Additional file 16: Table S10. Top bio functions and top canonical pathways of the Venn diagram. Table S10 exhibits the list of top bio functions or pathways of the Venn diagram for four different cancer types. Grey cells show the most significant functions/pathways at 5% FDR. (XLSX 18 kb)

## Abbreviations

aCGH: Array-comparative genomic hybridization
B: Breast
BEC: Breast, endometrial, cervix
BOC: Breast, ovarian, cervix
BOE: Breast, ovarian, endometrial
BOEC: Breast, ovarian, endometrial, cervix
C: Cervix
CBS: Circular binary segmentation
CNA: Copy number alteration
E: Endometrial
FDR: False discovery rate
GISTIC: Genomic Identification of Significant Targets in Cancer
HPV: Human papillomavirus
IPA: Ingenuity pathway analysis
O: Ovarian
OEC: Ovarian, Endometrial, Cervix (female reproductive system)
PCF: Piecewise Constant Fitting
REC: Regional Committees for Medical and Health Research Ethics
