# Correction to: A systematic comparison of copy number alterations in four types of female cancer

**DOI:** 10.1186/s12885-017-3766-7

**Published:** 2018-01-16

**Authors:** Fatemeh Kaveh, Lars O. Baumbusch, Daniel Nebdal, Anne-Lise Børresen-Dale, Ole Christian Lingjærde, Hege Edvardsen, Vessela N. Kristensen, Hiroko K. Solvang

**Affiliations:** 1 Department of Genetics, Institute for Cancer Research, Oslo University Hospital Radiumhospitalet, Oslo, Norway; 2 Medical Genetics Department, Oslo University Hospital Ullevål, Oslo, Norway; 3 Department of Pediatric Research, Division of Pediatric and Adolescent Medicine, Oslo University Hospital Rikshospitalet, Oslo, Norway; 4 Department of Computer Science, University of Oslo, Oslo, Norway; 5 Department of Clinical Molecular Biology (EpiGen), Medical Division, Akershus University Hospital, Lørenskog, Norway; 6 Marine Mammals Research Group, Institute of Marine Research, Bergen, Norway

## Correction

After publication of the original article [1] the authors found that the article contained an incorrect version of Fig. 4. This does not affect the results and conclusions of the article.

An updated version of Fig. 4 is included with this Correction.

**Fig. 4:**
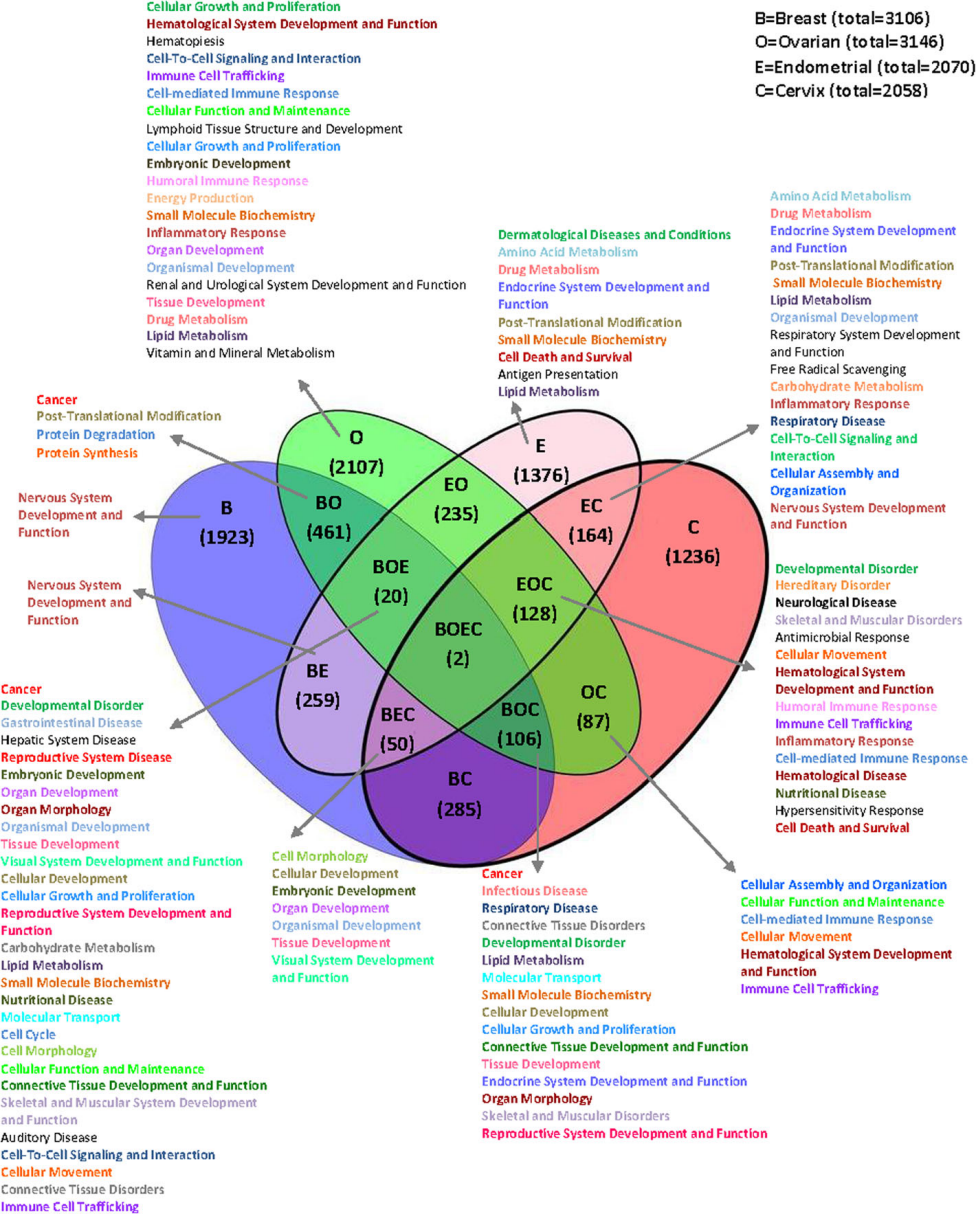
Overlap between gene sets of four female cancer– Top biological functions. The Venn diagram displays joint genes identified by both, CBS and PCF algorithms located within the regions identified by GISTIC. The total number of genes for each data set is presented on the top right panel. Top biological functions and top canonical pathways for each region of the overlapped cancers are stated
