# Supplementary material for: A systematic comparison of copy number alterations in four types of female cancer
Source: BMC Cancer. 2016 Nov 22;16:913. doi: 10.1186/s12885-016-2899-4 (PMC5120489; doi:10.1186/s12885-016-2899-4)
Supplement: Additional file 2: — Supplementary methods (DOCX 398 kb) [file 12885_2016_2899_MOESM2_ESM.docx]

# Supplementary methods

#### Various segmentation methods exist for the analysis of copy number data, and GISTIC output may to some degree depend on the choice of segmentation algorithm. The alterations of copy number data are highly associated with the development of different carcinomas which can lead to cancer development and shared features by the different cancer types. We propose a numerical method to obtain consistent GISTIC output across different segmentation methods. In this study, two segmentation methods, circular binary segmentation (CBS) and piecewise constant fitting (PCF) were considered.

## Circular binary segmentation (CBS)

#### CBS is the most commonly used algorithm for segmentation of copy number data. CBS is the modified version of binary segmentation that splits chromosomes into contiguous regions based on a maximum t-statistic estimated by permutation [1].

Let log ratio data $y_{1},y_{2},...,y_{n}$be ordered by the location of the probes on the chromosome. A break point corresponds to where the copy number changed on a chromosome. There is a change point if $y_{1},y_{2},\ldots,y_{v}\sim F_{0}$ and$y_{v+1},y_{v+2},\ldots,y_{n}\sim F_{1}$. CBS views the data as if on a circle and segment it into two arcs. Suppose$S_{i}=y_{1}+\ldots+y_{i}$ are the partial sums where$i=1,\ldots,n$. When the log ratio data are normally distributed, the likelihood ratio statistic for testing the null hypothesis $(H_{0}:\text{no change point} )$against the alternative$(H_{a}:\text{Change point at }v=i)$is given by where

In this study, we used the implementation of CBS found in the Bioconductor package “DNAcopy” [2]. The default values for the minimum number of markers for a changed segment (*min-width*) is 2 and the significant levels of test for accepting the change points (α) is 0.01.

## Piecewise Constant Fitting (PCF)

PCF is another segmentation algorithm for copy number data and is based on solving a penalized least squares regression problem [5]. Related to a Potts filter [3], a piecewise constant function is fitted to the data, and the parameters to be estimated are the number of break points and the genomic locations of the break points. Let log ratio data $y_{1},y_{2},...,y_{n}$be ordered by the location of the probes on the chromosome. Suppose that for each locus, the measurement is modeled as

$y_{j}=t_{j}+\varepsilon_{j}$ (1)

where $\varepsilon_{j}$represents the measurement noise. The log ratio points $y_{1},y_{2},\ldots,y_{n}$are to be fitted by corresponding parameters$t_{1},t_{2},\ldots,t_{n}$. A change point occurs between probes $j$ and $j+1$ if $t_{j}\neq t_{j+1}$ . Let $D=\left\{I_{1},\ldots,\left.I_{m-1}\right\}\right.$denote the segmentation of the chromosome arm induced by a particular change point sequence $t_{1},t_{2},\ldots,t_{n}$ . We fit the model in (1) by minimization of the penalized least squares criterion

$\sum_{j=1}^{n} (y_{j}-t_{j})^{2}+\gamma\cdot\left|D\right|$ (2)

The first term measures the distance between the observed log R ratios and the curve, while the second term is the penalty for change points; $\left.\left|D\right.\right|$ denotes the number of change points and$\gamma>0$ is a constant that controls the trade-off between the two terms. In this study, we used the implementation of PCF found in the Bioconductor package "copynumber" [5]. The user can specify $\gamma$ or use the default value of $\gamma=40$ which is often appropriate.

## Simulation study

We examined the results of applying two different segmentation methods followed by GISTIC 2.0 for identification of recurrent copy number alterations in a simulation study. We generated a simulation data set consisting of 100 samples. Let $u=\left\{(x_{ij},y_{ij}),i=1,\ldots,m,j=1,\ldots,\left.n\right\}\right.$be the copy number data, where $m$is the number of samples$(m=100)$ and $y_{i1},y_{i2},\ldots,y_{in}$are the log-ratio measurements of one sample that are ordered by the location of a probe along a chromosome ($x_{i1}\leq x_{i2}\leq\ldots\leq x_{in}$), where $n$ is the number of probes$(n=109302)$. In this simulation, the number of probes was determined from the actual annotation file (probe IDs and position) for the breast cancer data. In the first set of simulation, the minimum length of segments was equal to 50 and the maximum length was 700 probes. We generated the probes with the normal distribution (mean = 0) and the standard deviation of 0.10. To emulate the complexity of real tumour profiles, we added aberrations by uniform distribution on the interval between 0 and 1 that generates random deviates or aberrations. Any values less than 1 were set as a deletion and values greater than two was supposed being an amplification.

## Performance of CBS and PCF as input for GISTIC (Simulation study)

We segmented the simulation data by CBS and PCF for varying choices of the model parameters *α* in CBS and γ in PCF, and CBS and PCF segmented data were then used as input to GISTIC for identification of recurring copy number changes. Supplementary Table 2-A shows the number of focal peaks reported by GISTIC based on PCF segmentation. The number of focal peaks for the CBS segmented input data α varied, as shown in Supplementary Table 2-B. To identify proper thresholds for α and γ for the simulation data, we examined the overlap of GISTIC focal peaks obtained from CBS and PCF segmented data. Results were compared for both amplifications and deletions. The peaks that showed the highest percentage of overlap (over 70%) between CBS and PCF segmented data were selected as a likely result for α (CBS) and γ (PCF). GISTIC amplification and deletion peaks of simulation data showed over 93% of overlap between CBS and PCF. We observed that the highlighted values of α = 0.01 and γ = 16 demonstrate the most satisfactory and similar results for the simulated data. This result suggests that our proposed procedure can likely capture consistent GISTIC output for both CBS and PCF segmented data.

## Capturing consistent GISTIC output

To achieve consistent GISTIC output, we used segmented data from both CBS and PCF. These two algorithms were applied for segmentation of the DNA copy number data (breast, endometrial, ovarian and cervix). For CBS, we tested a range of different α values:

α = {0.00001, 0.00005, 0.0001, 0.0005, 0.001, 0.005, 0.01, 0.02,

0.03, 0.04, 0.05, 0.06, 0.07, 0.08, 0.09, 0.1}

For PCF segmentation, we tested a range of different γ values:

γ = {10, 11, 12, 13, 14, 15, 16, 17, 18, 20, 25, 30,

35, 40, 45, 50, 55, 60, 65, 70, 75, 80, 85, 90}

The parameter controlling the least allowed number of probes per segment (*min-width* in CBS and *kmin* in PCF) was set to 5.

## Performance of the overlap between CBS and PCF segmented data

Figure 1 shows the results from application of CBS and PCF for the selected values of α (0.02 for amplification and 0.05 for deletion) and γ (14 for both amplification and deletion) to the breast cancer data. The two top panels display the log ratio for sample 1 and 2 across the genome, with CBS segmentation shown as blue lines and PCF segmentation shown as red lines. The third panel zoomed into individual chromosome level (chromosome 1). We observed that selected values of α and γ illustrated a good overlap between CBS and PCF algorithms.


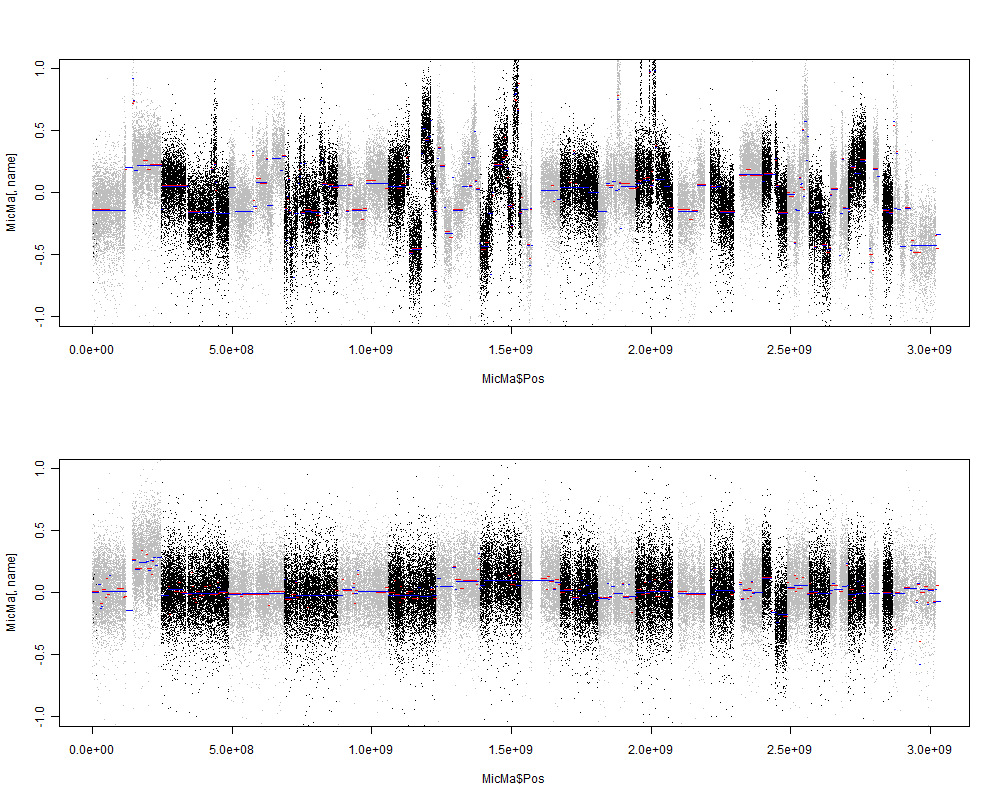


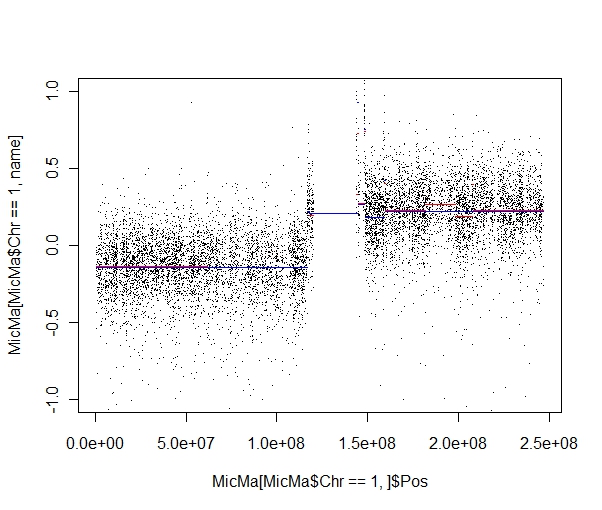

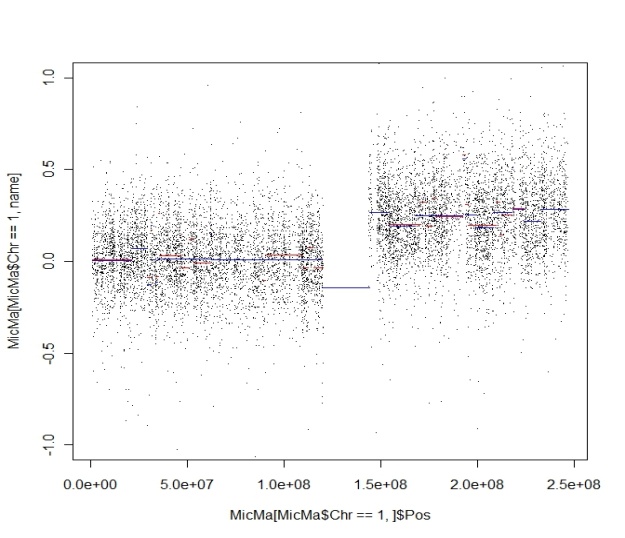


**Figure 1** illustrates the comparison of segmentation results using CBS and PCF on copy number data for genomic regions from breast cancer data samples no 1 and 2. CBS segmentation is shown in blue and PCF in Red. Two top panels show the log ratios of breast cancer data and the last panel shows the zoomed region of chromosome 1.

## Summary for consistent GISTIC outputs between CBS and PCF (real data)

We compiled high resolution copy number data from female cancer samples. We compared the application of two segmentation algorithms CBS and PCF by changing α and γ, respectively; and assessed the GISTIC 2.0 outputs to identify significant copy number alterations in real data.

Supplementary Table 3-A shows the number of obtained focal peaks while the input to GISTIC was PCF segmented. The number of focal peaks when the input data was CBS segmented and α varied, as highlighted in Supplementary Table 3-B.

Additional file 1, Figure S1 illustrates the number of peaks obtained by GISTIC, across the y-axis while changing two parameters; α (CBS) and $\gamma$(PCF) on the x axis. GISTIC peaks of amplification applying CBS segmented data are shown in pink and PCF segmented data in red, respectively. Deletion peaks are colored in green when the input is CBS segmented data and PCF segmented data in blue. Panels (A, C, E and G) highlight the GISITIC focal peaks for breast, ovarian, endometrial, and cervical cancers when input was PCF segmented data. Panels (B, D, F and H) illustrate the GISTIC focal peaks of the same data sets, while input was CBS segmented data. According to the GISTIC focal peaks (Supplementary Table 3-A and B), the similar numbers of CBS and PCF segmented data of each cohort were compared together for both amplification and deletion. Subsequently the highest percentage of overlap between CBS and PCF segmented data was selected as an identical result for α (CBS) and γ (PCF) (Supplementary Table 4). GISTIC amplification peaks of female cohorts showed over 80% of overlap between CBS and PCF algorithms (Supplementary Table 4-A). We observed that highlighted values of α = {0.02, 0.02, 0.02, 0.01} and γ = {14, 12, 14, 16} demonstrated the most satisfaction identical results for breast, ovarian, endometrial and cervical cancers, respectively. For GISTIC deletion peaks, α = {0.05, 0.05, 0.02, 0.005} and γ = {14, 13, 16, 25} illustrated the highest overlap for the female cohorts (Supplementary Table 4-B).

For the breast cohort, GISTIC peaks showed over 84% amplification and 69% deletion overlap between CBS segmented data (α = 0.02 for amplification and α = 0.05 for deletion) and PCF segmented data (γ = 14 for both amplification and deletion). CBS (α = 0.02 for amplification and α = 0.05 for deletion) and PCF (γ = 12 for amplification and γ = 13 for deletion) segmented of ovarian data showed 84% amplification and over 71% deletion overlap. Endometrial cohort exhibited over 82% amplification and deletion overlap between CBS (α = 0.02 for both amplification and deletion) and PCF (γ = 14 for amplification and γ = 16 for deletion) segmented data. We observed over 80% amplification and 58% deletion overlap between CBS (α = 0.01 for amplification and α = 0.005 for deletion) and PCF (γ = 16 for amplification and γ = 25 for deletion) cervical segmented data (Supplementary Table 4-A and B).

Comparison of GISTIC focal amplification peaks between four different cohorts and platforms revealed a good overlap when we chose α = 0.01 and 0.02 for CBS segmented input and γ = 12 till 16 for PCF segmented input. GISTIC focal deletion peaks showed that α varied between 0.005 till 0.05 while γ was changing from 13 till 25. This larger variation may be explained by the possibility of multiple copies for gains and less variations for deletions.

# Reference List

1. Olshen AB, Venkatraman ES, Lucito R, Wigler M: **Circular binary segmentation for the analysis of array-based DNA copy number data**. *Biostatistics* 2004, **5:**557-572.

2. Venkatraman ES, Olshen AB: **A faster circular binary segmentation algorithm for the analysis of array CGH data**. *Bioinformatics* 2007, **23:**657-663.

3. Winkler G, Liebscher V: **Smoothers for Discontinuous Signals**. *Journal of Nonparametric Statistics* 2002, **14:**203-222.

4. Lai WR, Johnson MD, Kucherlapati R, Park PJ: **Comparative analysis of algorithms for identifying amplifications and deletions in array CGH data**. *Bioinformatics* 2005, **21:**3763-3770.

5. Nilsen G, Liestøl K, Van Loo P, Moen Vollen HK, Eide MB, Rueda OM, Chin SF, Russell R, Baumbusch LO, Caldas C, Børresen-Dale AL, Lingjærde OC: **Copynumber: Efficient algorithms for single- and multi-track copy number segmentation.** *BMC Genomics* 2012, **13**:591.
