# Supplementary figures and images for: A systematic comparison of copy number alterations in four types of female cancer
Source: BMC Cancer. 2016 Nov 22;16:913. doi: 10.1186/s12885-016-2899-4 (PMC5120489; doi:10.1186/s12885-016-2899-4)

Additional file 1, Figure S1 - GISTIC outputs for CBS - or PCF - segmented input data.

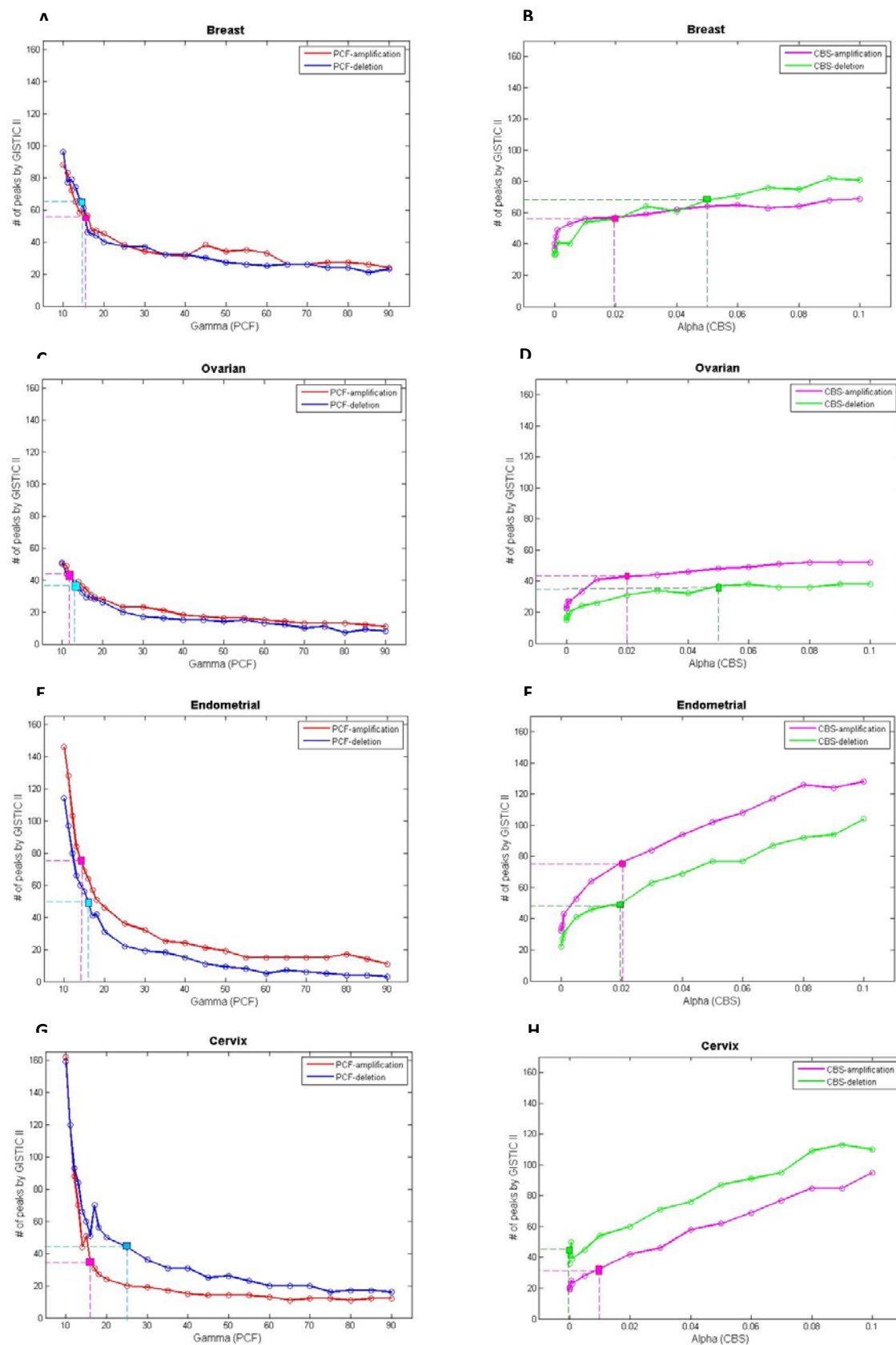

Supplement: Additional file 6: Figure S1. — Genomic Identification of Significant Targets (GISTIC) outputs for Circular Binary Segmentation (CBS) - or Piecewise Constant Fit (PCF) - segmented input data. The number of peaks attained by GISTIC on the y-axis is plotted against the two changing parameters α for CBS and γ for PCF on the x-axis. GISTIC peaks of amplification applying CBS-segmented data are illustrated in pink and PCF-segmented data in red, respectively. Deletion peaks are colored in green for CBS-segmented input data and in blue for PCF-segmented data. From top to bottom are shown GISTIC focal peaks for breast, ovarian, endometrial, and cervical cancers, to the left for PCF-segmented input data (A, C, E, and G) and to the right for CBS-segmented input data (B, D, F and H), respectively. For further analysis are the selected α and γ highlighted with a colored square. (PDF 362 kb) [file 12885_2016_2899_MOESM6_ESM.pdf]
