# Supplementary material for: A systematic comparison of copy number alterations in four types of female cancer
Source: BMC Cancer. 2016 Nov 22;16:913. doi: 10.1186/s12885-016-2899-4 (PMC5120489; doi:10.1186/s12885-016-2899-4)

Additional file 2, Figure S2 - Comparison of PCF and CBS results for detection of joint peaks in female cancers

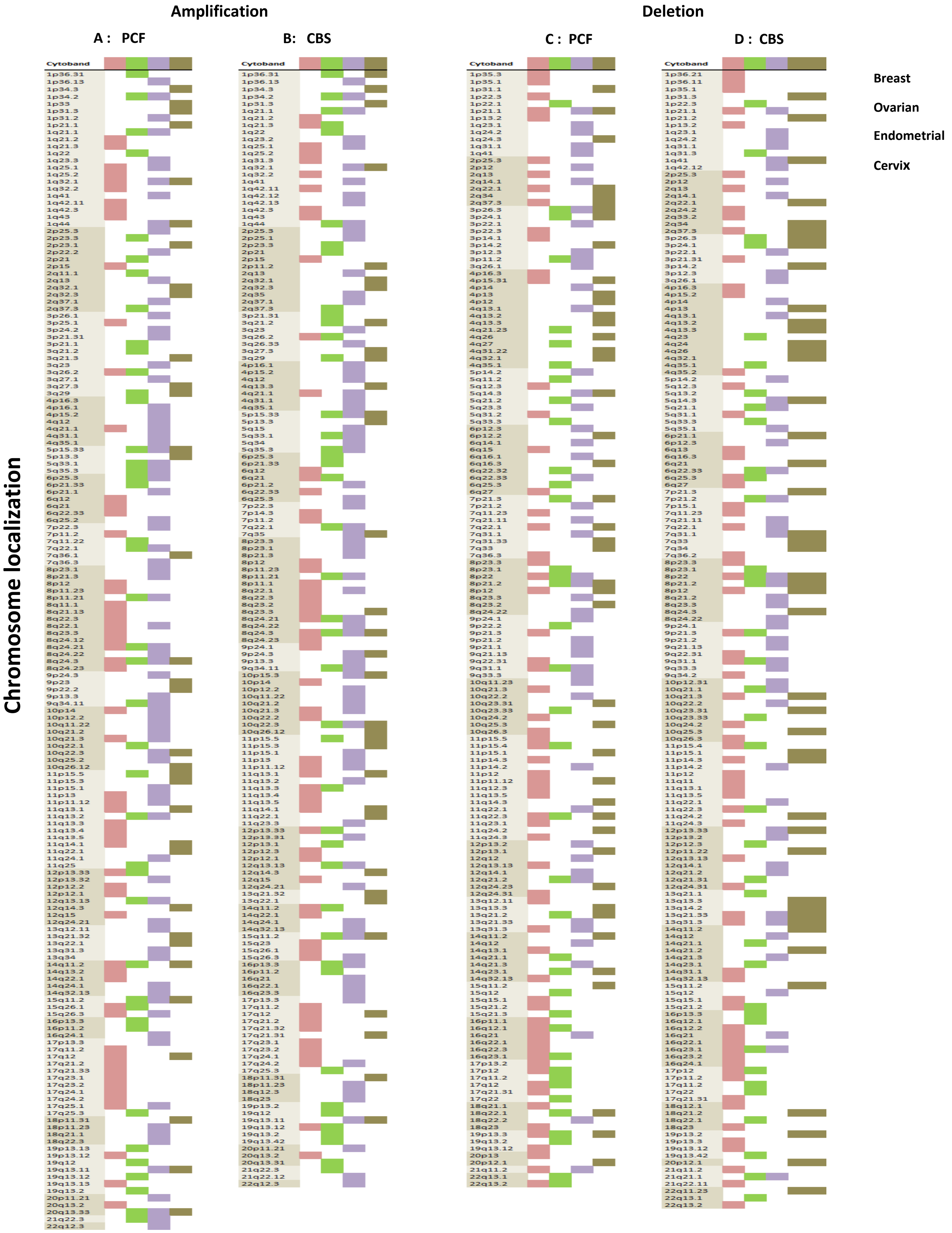

Supplement: Additional file 8: Figure S2. — Comparison of PCF and CBS results for detection of joint peaks in different female cancers. The number of peaks obtained by GISTIC are revealed for breast, ovarian, endometrial, and cervical cancers, colored in pink, green, purple, and brown, respectively. Panel A shows the amplification GISTIC focal peaks for PCF-segmented data and panel B for CBS-segmented input data. Panels C and D illustrate the GISTIC focal peaks for deletions for PCF- and CBS-segmented input data, respectively. (PDF 114 kb) [file 12885_2016_2899_MOESM8_ESM.pdf]
