# Supplementary material for: A systematic comparison of copy number alterations in four types of female cancer
Source: BMC Cancer. 2016 Nov 22;16:913. doi: 10.1186/s12885-016-2899-4 (PMC5120489; doi:10.1186/s12885-016-2899-4)

Additional file 3, Figure S3 - Focal peaks of GISTIC for female cancers based on CBS-segmented data (amplifications and deletions)

A

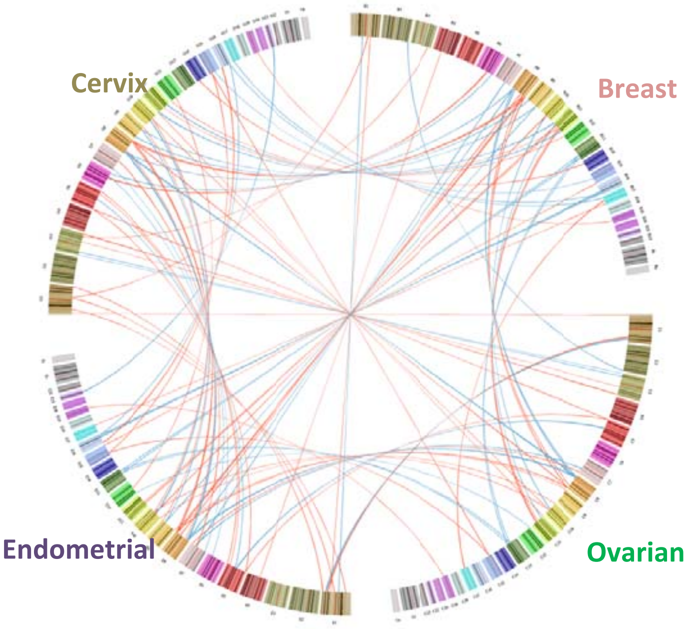

B

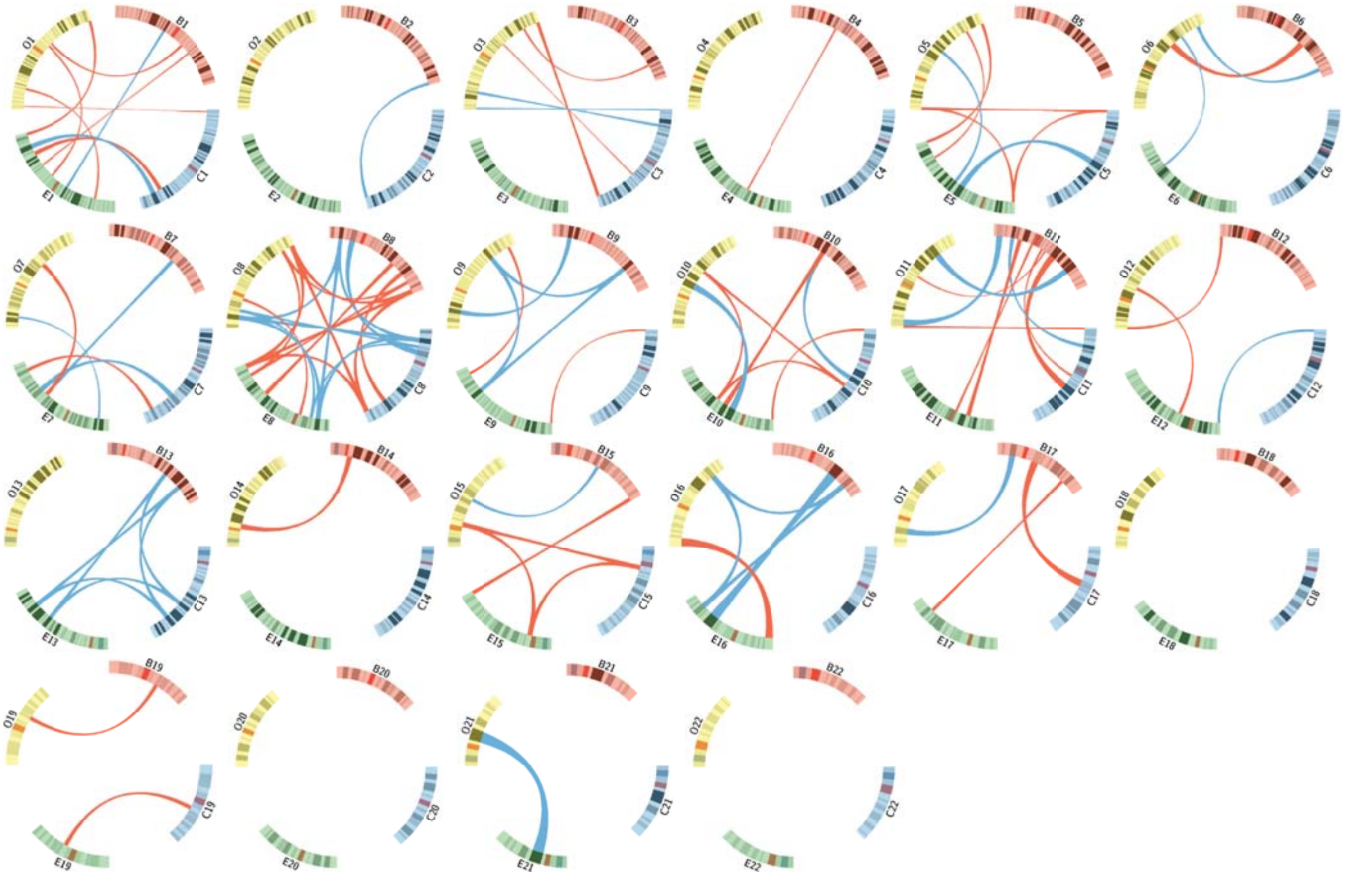

Supplement: Additional file 9: Figure S3. — Focal peaks of GISTIC for female cancers based on CBS-segmented data (amplifications and deletions). Panel A represents the aberrations of various female cancers for CBS-segmented input data to GISTIC in a circos-plot. In clockwise direction, breast (B, pink), ovarian (O, green), endometrial (E, purple), or cervical (C, brown) cancers are displayed. From the top of circles with 23 chromosomes presented for each cohort with each chromosome’s cytobands is colored differently. Aberrations are represented by lines linking the overlapping cytobands between the various female cancers. The width of lines is matched to the size of each cytoband. Amplification lines are colored in red and deletion lines are illustrated in blue. Panel B focuses separately on each chromosome. (PDF 329 kb) [file 12885_2016_2899_MOESM9_ESM.pdf]
