# Supplementary material for: A systematic comparison of copy number alterations in four types of female cancer
Source: BMC Cancer. 2016 Nov 22;16:913. doi: 10.1186/s12885-016-2899-4 (PMC5120489; doi:10.1186/s12885-016-2899-4)

Additional file 4, Figure S4 - Focal peaks of GISTIC for female cancers based on PCF-segmented input data (amplifications and deletions)

A

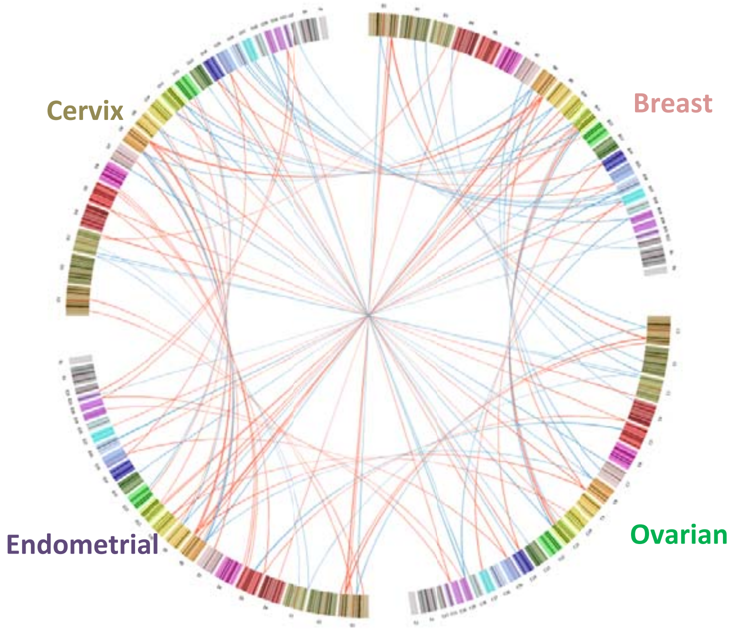

B

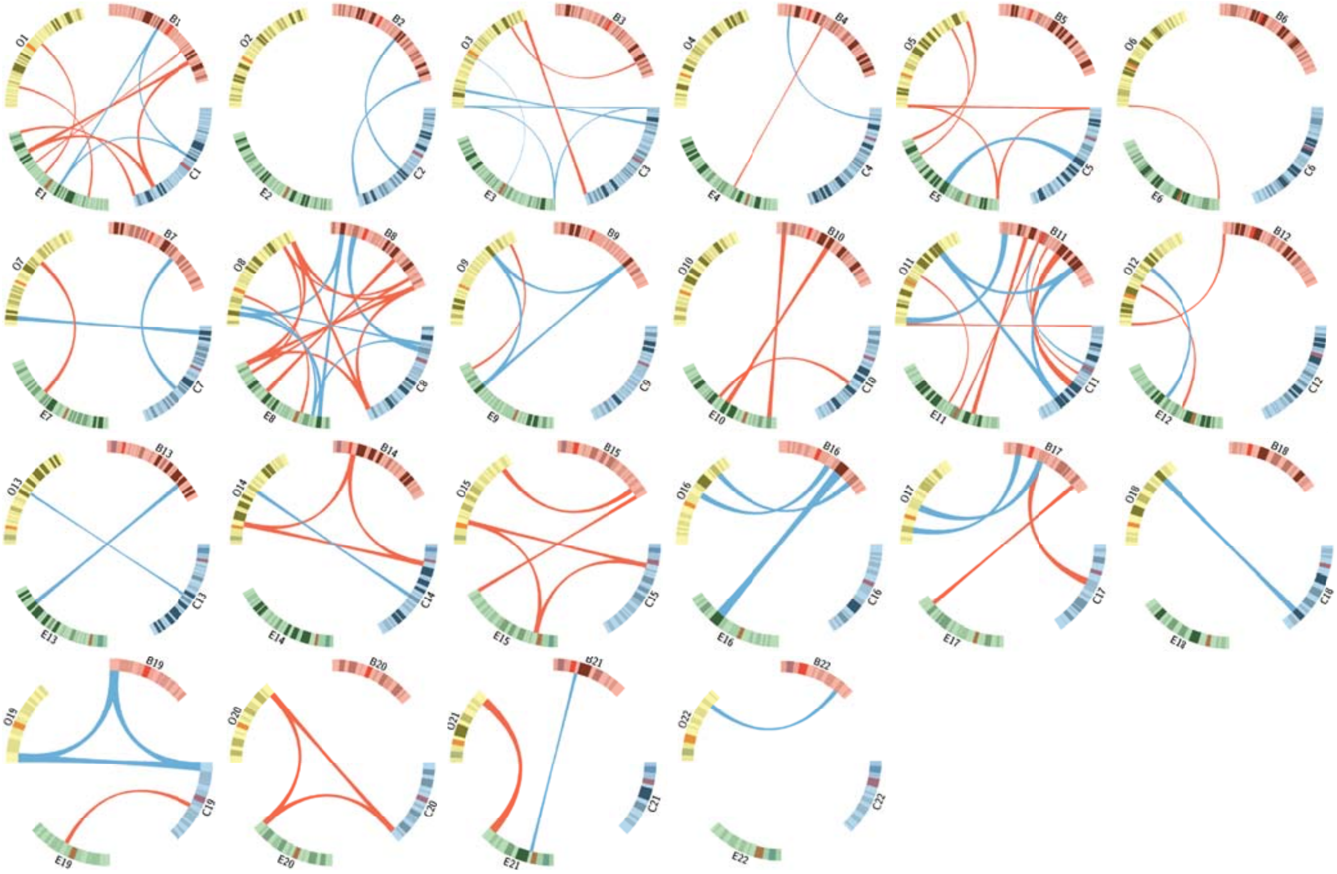

Supplement: Additional file 10: Figure S4. — Focal peaks of GISTIC for female cancers based on PCF-segmented input data (amplifications and deletions). Panel A represents the aberrations of female cancers in a circular format for PCF-segmented input data to GISTIC. In clockwise direction, breast, ovarian, endometrial, and cervical cancers are displayed in pink (breast), green (ovarian), purple (endometrial), or brown (cervical) cancers. From top of the circles are displayed 23 chromosomes for each cohort, each chromosome’s cytobands colored differently. Rearrangements are represented by lines connecting the overlapping cytobands between the different female cancers. The width of lines is matched with the size of each cytoband. Amplification lines are colored in red and deletion lines in blue, respectively. Panel B focuses separately on each chromosome. (PDF 330 kb) [file 12885_2016_2899_MOESM10_ESM.pdf]
