# Supplementary material for: A systematic comparison of copy number alterations in four types of female cancer
Source: BMC Cancer. 2016 Nov 22;16:913. doi: 10.1186/s12885-016-2899-4 (PMC5120489; doi:10.1186/s12885-016-2899-4)

Additional file 5, Figure S5 - A schematic view of genomic position of two common genes among female cancers

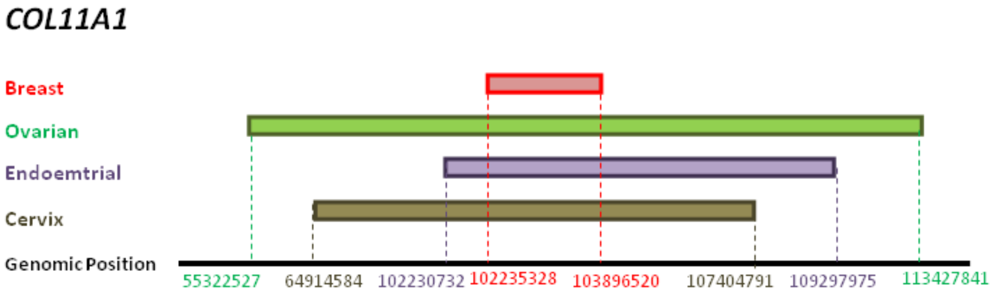

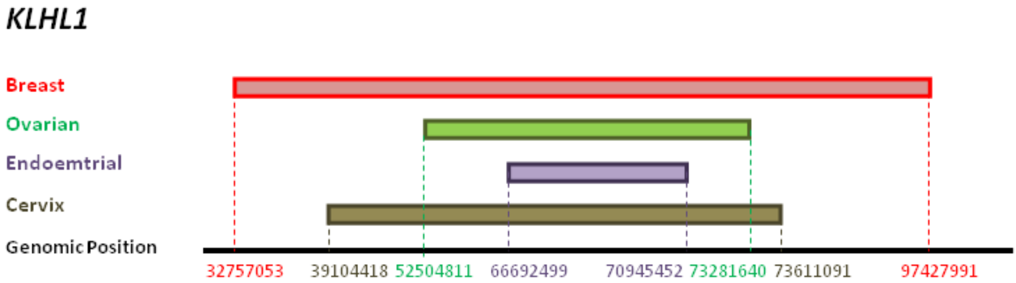

Supplement: Additional file 13: Table S8. — Genes residing in loci of specific amplifications and deletions (CBS and PCF) - gains and losses. Table S8. reveals the list of genes for the selected values of α (CBS) and γ (PCF). (PDF 41 kb) [file 12885_2016_2899_MOESM13_ESM.pdf]
